# Assessment of plasma Catestatin in COVID-19 reveals a hitherto unknown inflammatory activity with impact on morbidity-mortality

**DOI:** 10.3389/fimmu.2022.985472

**Published:** 2022-09-29

**Authors:** Francis Schneider, Pierrick Le Borgne, Jean-Etienne Herbrecht, François Danion, Morgane Solis, Sophie Hellé, Cosette Betscha, Raphaël Clere-Jehl, François Lefebvre, Vincent Castelain, Yannick Goumon, Marie-Hélène Metz-Boutigue

**Affiliations:** ^1^ Médecine Intensive Réanimation, Hôpital de Hautepierre, Hôpitaux Universitaires de Strasbourg, Fédération de Médecine Translationnelle de Strasbourg (FMTS) and Unistra, Strasbourg, France; ^2^ Institut National de la Santé et de la Recherche Médicale-Unité Mixte de Recherche (INSERM-UMR) 1121 Biomatériaux et Bio-ingénierie, Fédération de Médecine Translationnelle de Strasbourg (FMTS) and Unistra, Strasbourg, France; ^3^ Service d’accueil des urgences, Hôpital de Hautepierre, Hôpitaux Universitaires de Strasbourg, Fédération de Médecine Translationnelle de Strasbourg (FMTS) and Unistra, Strasbourg, France; ^4^ Maladies Infectieuses et Tropicales, Nouvel Hôpital Civil, Hôpitaux Universitaires de Strasbourg, Strasbourg, France; ^5^ Laboratoire de Virologie, Hôpitaux Universitaires de Strasbourg, Fédération de Médecine Translationnelle de Strasbourg (FMTS), Faculté de Médecine and Unistra, Strasbourg, France; ^6^ Pôle de Santé Publique, Groupe de Méthodes en Recheche Clinique (GRMC), Hôpitaux Universitaires de Strasbourg, Unistra, Strasbourg, France; ^7^ Centre National de la Recherche Scientifique-Unité Propre de Recherche (CNRS-UPR) 3212, Institut des Neurosciences Cellulaires et Intégratives, Unistra, Strasbourg, France

**Keywords:** Innate immunity, COVID, Catestatin, Chromogranin A, hypoxia, critically ill, nosocomial disease

## Abstract

**Introduction:**

Neuroendocrine cells release Catestatin (CST) from Chromogranin A (CgA) to regulate stress responses. As regards COVID-19 patients (COVID+) requiring oxygen supply, to date nobody has studied CST as a potential mediator in the regulation of immunity.

**Patients & Methods:**

Admission plasma CST and CgA - its precursor - concentrations were measured (ELISA test) in 73 COVID+ and 27 controls. Relationships with demographics, comorbidities, disease severity and outcomes were analysed (Mann-Whitney, Spearman correlation tests, ROC curves).

**Results:**

Among COVID+, 49 required ICU-admission (COVID+ICU+) and 24 standard hospitalization (COVID+ICU-). Controls were either healthy staff (COVID-ICU-, n=11) or (COVID-ICU+, patients n=16). Median plasma CST were higher in COVID+ than in controls (1.6 [1.02; 3.79] *vs* 0.87 [0.59; 2.21] ng/mL, *p*<0.03), with no difference between COVID+ and COVID-ICU+. There was no difference between groups in either CgA or CST/CgA ratios, but these parameters were lower in healthy controls (*p*<0.01). CST did not correlate with either hypoxia- or usual inflammation-related parameters. In-hospital mortality was similar whether COVID+ or not, but COVID+ had longer oxygen support and more complications (*p*<0.03). CST concentrations and the CST/CgA ratio were associated with in-hospital mortality (*p*<0.01) in COVID+, whereas CgA was not. CgA correlated with care-related infections (*p*<0.001).

**Conclusion:**

Respiratory COVID patients release significant amounts of CST in the plasma making this protein widely available for the neural regulation of immunity. If confirmed prospectively, plasma CST will reliably help in predicting in-hospital mortality, whereas CgA will facilitate the detection of patients prone to care-related infections.

## Introduction

Catestatin (CST, human CgA352-372) is naturally produced from chromogranin A (CgA) in nervous, endocrine and immune cells by the action of several proteolytic enzymes, such as cathepsin L (1), plasmin (2, 3) and furin in addition to PC1/PC2 prohormone convertases and thrombin (4). The prohormone thiol protease (PTP) is also essential for CST production (5, 6). CST is an endogenous inhibitor of the nicotinic cholinergic receptor (6–8) with several biological activities: modulation of inflammation (9, 10) - including activation of polymorphonuclear white blood cells (PMNs) (11) and of mast cells (12)-, antimicrobial activities (13, 14), and homeostatic and metabolic regulations (15). CST, which penetrates in several immune cells lines (12, 16), may also be involved in the occurrence, amplification and/or regulation of severe inflammatory conditions resembling those occurring during COVID. Literature suggests CST be targeted for treatment of inflammatory diseases (17). To date, plasma CST concentrations have not been assessed in SARS-CoV-2 infection (COVID-19) or in non-COVID intensive care unit (ICU) patients with systemic inflammation. Recently, a publication has reported that increased plasma concentration of its precursor CgA predicts mortality in some COVID patients (18).

Clinical aspects of acute COVID-19 range unpredictably from those of a mild flu-like disease to fatal multiple organ failure (19). For example, pulmonary parenchymal injuries can progress from acute lung injury to fatal acute respiratory distress syndrome in a context of intractable inflammation even in the absence of documented risk factors for COVID (20, 21). Whether serious inflammatory aggravation after contamination is reliably foreseeable for any given patient is not known. Pre-existing cardiovascular and respiratory disorders, diabetes, obesity, cancer and immune dysregulation (22, 23) worsen sensitivity to the virus, and a negative outcome is often associated with increased levels of C-reactive protein as well as with intractable white blood cells activation in COVID (22). This includes an imbalanced innate host response to the SARS-CoV-2 with an aberrant activation of PMNs (23), and with unusually high release of pro-inflammatory cytokines (24). A recently reported comparison of circulatory levels of neutrophils secretory proteins showed that defensin DEFA1 level is higher in severe than in mild COVID patients (25, 26). Similar data were published for the antimicrobial peptide LL-37 (26).

The present study was conducted on the hypothesis that the CgA- derived antimicrobial peptide CST is involved in the pathophysiology of the severe SARS-COVID-2-driven inflammation for two reasons. Firstly, CgA–related peptides are detected in the blood of patients suffering severe systemic inflammation (27). Secondly, *ex vivo* CST significantly stimulates the release by human PMNs of many innate-immunity-associated factors, including S100 calcium binding proteins (11). In our study, we checked whether admission measurement of CST and its precursor CgA might help clinicians in understanding and assessing the risk of severe evolution in COVID among patients admitted to an emergency department with acute respiratory failure requiring immediate oxygen supply.

## Materials and methods

The Ethical Committee of our institution approved this study. All participants gave informed consent.

### Definitions

Participants were classified COVID+ in the presence of a clear diagnosis of COVID (*i.e.*: positive rt-PCR with suggestive CT-scan) and, conversely, COVID- in all other cases. Controls were always COVID-, but were either healthy staff (therefore called COVID-ICU-) or multiple organ failure patients requiring ICU admission (COVID-ICU+).

### Patients

We recruited non-vaccinated participants in the emergency department during the first two surges of the COVID pandemic. Inclusion criteria were age over 18 years, clinical features of acute-onset respiratory disease (SpO_2_<90% in the absence of oxygen supply), with a low dose CT-scan of the chest typical of COVID, and nasal (or lower respiratory tract) swab with a rt-PCR positive for SARS-CoV-2. Whether a patient needed a transfer to the ICU was decided if hypoxemia persisted despite 15 L/min-nasal oxygen supply during 1h without relief of respiratory failure. Conversely, if relieved by low inspired fraction of oxygen, patients were admitted to the department for infectious diseases. Exclusion criteria were interfering causes for increased circulating CgA. Alternatively, controls were either healthy members of staff (COVID-ICU-) or ICU-patients with neither criteria for COVID, but at least one life-threatening organ failure (COVID-ICU+).

We recorded many variables on admission and thereafter: age, gender, body mass index (BMI), time from first symptom to hospital admission, comorbidities (tobacco smoking, chronic renal failure, diabetes, hypertension, diseases with active immune suppression – *i.e.*: organ transplantation, chemotherapy-induced neutropenia-, chronic liver failure). We also assessed standard admission biological parameters: biochemistry with arterial PaO_2_, PaO_2_/FiO_2_, lactate, and inflammation parameters, such as admission and maximum (3^rd^ day) C-reactive protein, ferritin and haematology tests (leucocytes counts and coagulation factors). Severity of ICU+ patients was defined with the SAPS II score (28). All patients were prescribed oxygen supply. Organ support characteristics are reported for ICU-patients: respiratory support (FiO_2_, ventilation support, duration of support, care-related infections), complications (pulmonary embolism, stroke, acute circulatory failure), specific treatments (requirement for norepinephrine, extracorporeal membrane oxygenation, haemodialysis….). Finally, we measured outcome parameters with attention paid to the occurrence of care-related infections, and to mortality within the ICU and in-hospital.

### SARS-CoV-2 detection

Respiratory samples (i) underwent extraction using the eMAG^®^/eSTREAM^®^ system (bioMérieux, Marcy l’Etoile, France) followed by amplification on the LightCycler^®^ 480 Instrument II (Roche Diagnostics, France) for the first wave, and (ii) were extracted and amplified on the Hologic Panther Fusion^®^ system for the second wave. We performed real-time RT-PCR targeting the RdRp gene to test positivity for the SARS-CoV2 virus. We used the Pasteur Institute multiplexed primers and probe sets Flo2 and Flo4: CoV_IP2-12669Fw ATGAGCTTAGTCCTGTTG and CoV_IP2-12759Rv CTCCCTTTGTTGTGTTGT with probe CoV_IP2-12696Probe(+) AGATGTCTTGTGCTGCCGGTA[5’]Hex[3’]BHQ-1; CoV_IP4-14059Fw GGTAACTGGTATGATTTCG, and CoV_IP4-14146Rv CTGGTCAAGGTTAATATAGG with probe CoV_IP4-14084Probe(+) TCATACAAACCACGCCAGG[5’]Fam[3’]BHQ-1. We used RNA copies dilutions of the CoV_ IP transcript to assess equivalence between cycle threshold values and quantitation in copies/reaction for the CoV_IP4 target.

We performed diagnostic low-dose CT scans of the chest in accordance with recommendations (29).

### Dosages of chromogranins

At admission, we performed dosages of both CgA and CST in the plasma using commercially available ELISA kits with the manufacturer’s instructions (Biomatik, Kitchener, Ontario, Canada). For CST, the minimum detection limit of the kit was 0.078 ng/mL and the detection range ranged from 0.312 ng/mL to 20 ng/mL (kit EKC33038-96T). For CgA, the minimum detection limit was 13.7 pg/mL and the detection ranged from 31.2 pg/mL to 2,000 pg/mL (EKU03179-96T). Samples were prepared after a dilution of serum in PBS of 1/100 for CgA and 1/50 for CST. We performed each analysis in triplicate. We calculated the amounts of both CST and CgA on basis of the dilution used and with standard curves.

### Statistical analysis

The statistical analyses included a descriptive section and an analytical section. For categorical variables, the descriptive analysis was performed giving counts and percentages; for continuous variables giving medians and 1^st^ - 3^rd^ quartiles. We tested distribution normality using the Shapiro–Wilk test. Comparisons between categorical variables were performed using the Chi-squared test or Fisher’s exact were expected values in any of the cells of the contingency table were below 5. Dot Plot was obtained by using GraphPad Prism 6 Software.

Comparisons between continuous and categorical variables were performed using the Student’s t-test (or ANOVA) or Wilcoxon’s test (or the Kruskall-Wallis test) in case of heteroscedasticity or if the variable did not follow a normal distribution. We tested the associations between two continuous variables with the Spearman’s rank correlation rho.

Receiver operating characteristic (ROC) curves were plotted and the areas under the receiver operating characteristic curve (AUC) were estimated with sensitivity, specificity, positive and negative predictive values for the best cut-off estimated with the Youden index. The significance level was set at 5%. Analyses were performed with R 4.0.2 software.

## Results

### Study population characteristics

**Figure 1:**
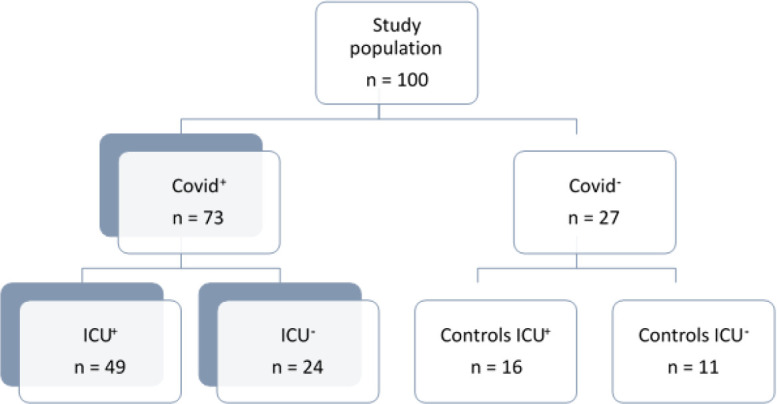
Flow chart of the study. Study participants (n=100) were screened for participation during the first surge of the disease (March to May 2020) among 547 patients admitted for COVID either to the emergency department or to the ICU. Informed consent for participation was obtained in 49 COVID+ ICU+ patients, and in 24 COVID+ICU- patients, which were then admitted to the infectious disease department. In parallel, 11 participants were recruited in our staff (as healthy controls, COVID-ICU-), and so were 16 COVID-ICU+ patients that were admitted for non-COVID multiple organ failure requiring mechanical ventilation support.

100 participants (36 women and 64 men) were included over 2 months in 2020; respiratory samples were COVID+ in 73 patients and COVID- in 27 controls (**Figure 1**). COVID-ICU+ (n=16) were mainly septic shock patients. **Tables 1** and **2** present the clinical features and hospital stay characteristics. Briefly, 36 women and 64 men were involved. Healthy controls were significantly younger than both COVID+ICU+ patients and COVID-ICU+ controls. 26% of the participants – but not healthy controls - were obese (BMI > 30); the BMI was higher in COVID+ patients than in controls (*p*<0.001). COVID+ICU+ patients were less severe than COVID-ICU+ controls according to the SAPS II score (*p*<0.03). Comorbidities were significantly more frequent in COVID+ and ICU+ patients. ICU+ COVID+ patients had significantly less acute renal failure than did COVID- ICU+ (*p*<0.03). As indicated (**Table 1**), COVID+ patients had severe hypoxemia with hyper-inflammatory profiles as compared with healthy controls. However, we failed to detect any significant correlation between (i) PaO_2_, the PaO_2_/FiO_2_ ratio or inflammatory parameters (CRP, ferritin, fibrinogen) and (ii) CST or CgA (this in any or all groups of participants). As expected, ICU+ patients required significant respiratory, circulatory and renal support as compared with controls. Only COVID+ICU+ patients were treated with steroids, and this after CST and CgA initial assessment; the duration of mechanical ventilation support was nevertheless three-times longer in COVID+ ICU+ than in their controls (*p*<0.001).

**Table 1:** Characteristics of the study population at inclusion. Bold values correspond to significative *p* value.

		All participantsn = 100	COVID+ (n = 73)		Controls (n = 27)		*p* value
			ICU+n = 49	ICU-n = 24	COVID - ICU+(ICU Controls)n = 16	COVID - ICU-Healthy Controlsn = 11	
	Age (years), median [IQR1; IQR3]	70 [56; 78]	70 [63; 79]	77 [70; 86]	66 [59; 76]	36 [33; 45]	**<0.001**
	Gender, Male/Female (%)	64/36	35/14	11/13	10/6	8/3	0.178
	BMI (kg/m^2^) median [IQR1; IQR3]	27.2 [24.1; 30]	28 [25; 31]	27.9 [24.5; 31.1]	24.5 [22.1; 27.9]	23 [22.0; 23.5]	**<0.001**
	SAPS II median [IQR1; IQR3]	45 [40; 58]	44 [37; 54]	NR	54.5 [44.75; 65.5]	NR	**<0.03**
Comorbidities	Chronic renal failure n (%)	12 (12.0)	3 (6.1)	4 (16.7)	5 (31.3)	0 (0.0)	**0.027**
	Type II diabetes n (%)	30 (30.0)	12 (24.5)	10 (41.7)	8 (50.0)	0 (0.0)	**0.013**
	Hypertension n (%)	44 (44.0)	22 (44.9)	15 (62.5)	7 (43.8)	0 (0.0)	**0.004**
	Active smoking n (%)	15 (15.0)	3 (6.1)	2 (8.3)	5 (31.3)	5 (45.5)	**0.002**
	Chronic heart disease n (%)	30 (30.0)	12 (24.5)	11 (45.8)	7 (43.8)	0 (0.0)	**0.014**
	Immune suppression n (%)	14 (14.1)	8 (16.3)	1 (34.4)	5 (31.3)	0 (0.0)	0.064
	Cancer (< 5 years) n (%)	13 (13.1)	5 (10.2)	1 (4.4)	7 (43.8)	0 (0.0)	**0.003**
	Liver cirrhosis (n, %)	6 (6.1)	1 (2.0)	1 (4.4)	4 (25.0)	0 (0.0)	**0.021**
Biological parameters	rt-PCR (log copies/reaction)	5.33 [4.19; 6.30]	4.88 [4.03; 6.07]	5.67 [5.1; 6.62]	0	0	0.192
	Glycaemia (g/L)	1.25 [1.00; 1.55]	1.44 [1.14; 2.14]	1.04 [0.9; 1.3] ^&^	1.23 [1.14; 1.37]	0.84 [0.77; 0.94]^#^	**<0.001**
	C- reactive protein (mg/L)	110 [50.0; 180.9]	153.3 [88.0; 197.6]	48.0 [20.0; 75.0]	57.4 [31.0; 104.3]	NR	**<0.001**
	Maximal C- reactive protein (mg/L)	188.6 [102.7; 272.7]	222.9 [120.9; 282.8]	na	102.4 [57.8; 176.1]	NR	**<0.001**
	High sensitivity Ic Troponin (ng/L)	58.3 [28.7; 232.0]	52.7 [20.0; 238.8]	na	80.6 [42.5; 160.7]	na	0.564
	Ferritin (µg/L)	1233 [624.5; 2771]	1187 [563.8; 1632.5]	na	2864 [1331; 3992]	na	0.057
	Albumine (g/L)	25.8 [22.2; 29.0]	25.0 [22.2; 27.5]	na	28.0 [22.8; 29.0]	na	0.619
	CST (ng/mL)	1.4 [0.79; 3.24]	1.8 [0.9; 4.1]	1.36 [1.1; 1.86]	1.7 [0.97; 3.6]	0.62 [0.4; 0.75]	**<0.001**
			1.6 [1.02; 3.79]		0.87 [0.59; 2.21]		**0.027**
	CgA (ng/mL)	17.45 [11.2; 23.95]	17 [10.6; 25.3]	18.6 [17.3; 22.3]	19.7 [14.1; 35.7]	8.9 [7.7; 9.2]	**<0.001**
			18.5 [12.9; 23.5]		12.1 [9.0; 24.7]		0.086
	CST/CgA (%)	8.6 [4.2; 18.7]	11.6 [5.1; 32]	7 [3.2; 13.4]	8.7 [5.5; 16.9]	7.2 [4; 12.8]	0.148
			9.1 [4.3; 23.1]		8.2 [4.2; 12.7]		0.402
	CST/CgA (nM)	2.29 [1.05; 4.87]	3.19 [1.4; 6.34] 1.91 [0.87; 3.68]		2.29 [0.93; 3.74] 1.71 [0.87; 2.40		0.074
					
	White blood cells counts (G/mL)	7.70 [5.13; 11.59]	8.40 [5.30; 10.70]	4.68 [3.32; 6.28]	11.80 [7.80; 15.99]	na	**<0.001**
	Lymphocytes (%)	9.4 [4.9; 16.0]	8.6 [5.0; 15.2]	17.4 [14.5; 22.8]	5.9 [3.0; 13.4]	na	**0.007**
	Lactate (mmol/L)	1.15 [0.90; 1.80]	1.20 [0.90; 1.70]	1.00 [0.75; 1.35]	1.20 [0.95; 2.03]	na	0.608
	Creatinine (µmol/L)	71.0 [56.7; 113.9]	64.4 [55.1; 91.6]	95.3 [55.0; 105.1]	119.6 [72.0; 248.9]	na	<0.02
	D-Dimers (µg/L)	2100 [1130; 5450]	1900 [920; 4230]	na	4700 [2768; 7383]	na	0.051
	PaO_2_/FiO_2_	113 [75; 191]	95 [67; 126]	NR	285 [205; 334]	NR	**<0.001**
	Worse PaO_2_/FiO_2_	91 [74; 123]	82 [70; 101]	NR	207 [142; 280]	NR	**<0.001**
	PaCO_2_ (mm Hg)	NR	37.2 [33.2; 40.3]	NR	35.05 [30.8; 40.45]		**0.02**
Treatments	Mechanical ventilation support n (%)	55 (84.6)	42 (85.7)	NR	13 (81.3)	NR	0.936
	Ventilation (days) median [IQR1; IQR3]	10 [6; 17]	14 [8; 20]	NR	4.5 [2; 6]	NR	**<0.001**
	Norepinephrine infusion n (%)	47 (72.3)	38 (77.6)	NR	9 (56.3)	NR	0.187
	Renal replacement therapy n (%)	17 (26.2)	10 (20.4)	NR	7 (43.8)	NR	0.100
	Dexamethasone (6mg/d/10d) n (%)	25 (28.1)	25 (51.0)	0 (0)	0 (0)	NR	**<0.001**

na, not available.

NR, not relevant.

BMI, body mass index.

CgA, chromogranin A.

CST, catestatin.

SAPS II, Simplified Acute Physiological Score two.

Outcome issues appear in **Table 2**. ICU lengths of stay did not differ between ICU patients whether COVID+ or not, but in COVID+ICU- hospital length of stay was shorter than in COVID+ICU+ patients (6.5 [2; 10] days *vs* 27 [17; 47], *p*<0.001). Death rates were similar in ICU+ patients whether COVID+ or COVID-, with no differences in withholding and withdrawing decisions. Thromboembolic events were as frequent in COVID+ICU+ as in COVID-ICU+; the ratios of care-related infections were balanced in these two ICU-populations treated in parallel, by the same staff.

**Table 2:** Outcome issues. Bold values correspond to significative *p* value.

		All participantsn = 100	COVID+ ICU+n = 49	COVID+ICU-n = 24	COVID - ICU+(ICU Controls)n = 16	*p* value
Outcome						
	ICU length of stay (days)	14.0 [10.0; 26.0]	15.0 [10.0; 26.0]	NR	11.0 [6.8; 18.5]	0.115
	Hospital length of stay (days)	20.0 [9.0; 37.0]	27.0 [17.0; 47.0]	6.5 [2.0; 10.0]	26.5 [15.0; 49.8]	**<0.001**
	ICU-mortality (%)	16 (24.6)	10 (20.4)	NR	6 (37.5)	0.297
	In-hospital mortality (%)	25 (28.1)	12 (24.5)	7 (29.2)	6 (37.5)	0.562
	Thromboembolic event within hospital: n (%)	13 (14.6)	9 (18.4)	0 (0.0)	4 (25.0)	**0.028**
	Care-related infection: number of patients (%)	32 (36.0)	24 (49)	0 (0)	8 (50)	**<0.001**
	Causes of death (n, %)					
	- Refractory hypoxemia/lung fibrosis	8 (9.0)	1 (2)	7 (29.2)	0	**<0.001**
	- Multiple Organ Failure	15 (16.9)	9 (18.4)	0 (0)	6 (37.5)	**0.003**
	- Withdrawing & Withholding decisions	22 (24.7)	9 (18.4)	7 (29.2)	6 (37.5)	0.234

Data are either median [IQ1; IQ3]) or number (percentage, %).

NR, not relevant.

ICU, intensive care unit.

### CgA and CST plasma concentrations

**Figure 2:**
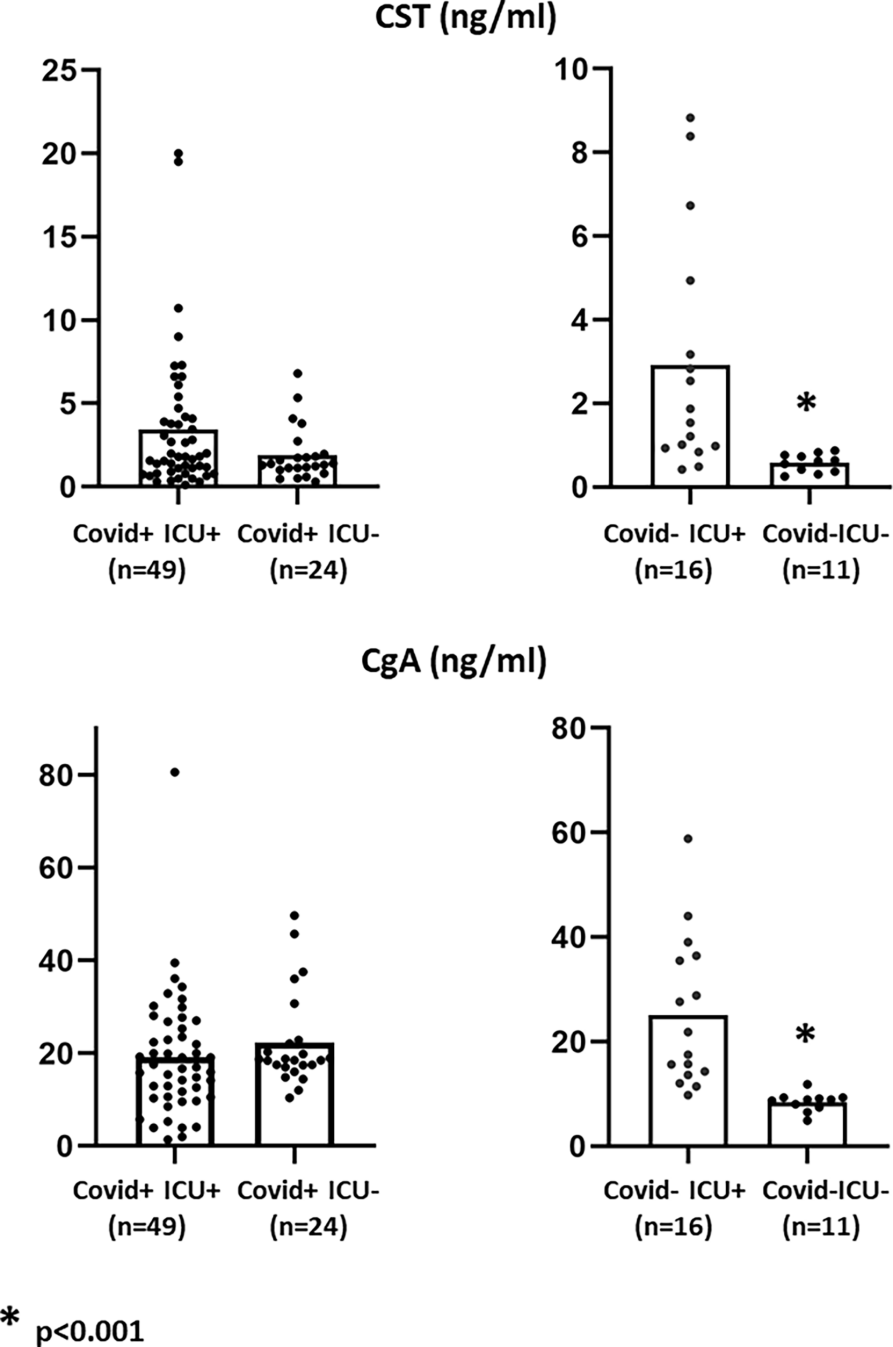
Admission concentrations of CST (top) and CgA (bottom) in the different study groups. On admission, whether COVID+ or not, patients had significantly (*p* < 0.001) higher plasma CST concentrations than healthy controls (COVID-, ICU-) without difference between COVID+ICU+ and COVID+ICU-. Admission CgA concentrations were decreased only in healthy controls.

As indicated (**Table 1** and **Figure 2**), COVID+ patients had significantly higher admission plasma concentrations of CST than COVID- participants (1.6 [1.02; 3.79] ng/mL *vs* 0.87 [0.59; 2.21] ng/mL, *p*= 0.027). In the global COVID- control study population, the levels of CgA were not significantly different between these two groups: 18.5 [12.9; 23.5] ng/mL *vs* 12.1 [9.0; 24.7] ng/mL, *p*=0.086). In fact, only healthy controls (COVID-ICU-) had significantly lower levels of both these peptides (*p*<0.001), with the other subgroups displaying comparable levels (**Figure 2**). Although there was a trend towards higher CST/CgA ratios in COVID+ICU+ patients as compared with others subgroups, the difference was not significant. Noteworthy is the fact that admission CST and CgA dosages were performed at the emergency department earlier after first symptoms onset in COVID+ICU- patients than in COVID+ICU+ ones (4[2.8; 7.0] days *vs* 8 [5; 12] days, *p*<0.007).

Finally, time-dependent dosages of CST and CgA were assessed daily over 72h in 2 subgroups of ICU+ patients, (*i.e.*: COVID+ICU+ (n=16) and COVID-ICU+ controls (n=5)) (**Figure S1**). During the first 2 days, CST tended to be lower in COVID+ as compared with COVID-, with an inverse trend over the following 2 days (**Figure S1**). CgA concentrations were stable in COVID+ patients (in the range of 20 ng/mL), and they were always higher in COVID- patients (26 ng/mL-34 ng/mL) with no significant time-dependent changes. Although the CST/CgA ratios were statistically similar, there was a trend towards a sharp increase in this ratio on the third day after ICU admission in COVID+ICU+ patients (this corresponds to the time-window with highest CRP concentrations at 3^rd^ day), whereas the ratio decreased in COVID-ICU+ controls (**Figure S1**).

### Associations between either CST, CgA, or the CST/CgA ratio and clinical data

CST and CgA concentrations never correlated with each other (whether in the study population as a whole or in any of the individual study subgroups). Both CST and the ratio CST/CgA were significantly associated with BMI >30 in all COVID+ patients (*p*<0.05). The presence of underlying type II diabetes was significantly associated with increased concentrations of CST (2.36 [1.19; 5.06] ng/mL *vs* 1.41 [0.80; 2.76], *p*=0.036); admission glycaemia correlated with CgA only in ICU+COVID+ patients (Spearman rho=0.284, n=49, *p*<0.05).

As far as outcome is concerned in COVID+ patients (n=73), CST concentrations and the CST/CgA were significantly associated with in-hospital mortality (*p*<0.01), whereas CgA concentrations were not (**Table 3**). Thus, the higher the concentration of CST on admission, the worse the outcome (1.36 [0.79; 2.49] ng/mL in survivors *vs* 2.82 [1.62; 5.99] ng/mL in non-survivor, (n=73, *p*= 0.0062)). However, among COVID+ICU+ patients (n=49), CST concentrations were 1.8 [0.8; 3.99] ng/mL in survivors *vs* 2.88 [1.59; 5.89] ng/mL in non-survivors (p=0.22), whereas in the COVID+ICU- participants (n=24), CST concentrations were 2.73 [1.62; 4.73] ng/mL in non-survivors *vs* 1.24 [0.80; 1.60] ng/mL in survivors (n=24, *p*= 0.009). These unexpected discrepancies led to the analysis of the CST/CgA ratios among subgroups. In COVID+ICU- (n=24), the lower the admission ratio CST/CgA, the better the outcome: 5.4 [3.0; 7.5]% in survivors *vs* 17.5 [12.6; 23.5]% in non-survivors (p= 0.019), whereas in the COVID+ICU+ group (n=49), there was no difference (17.7 [10.7; 80.8]% *vs* 9.4 [4.3; 21.7]%, (p= 0.12)).

**Table 3:** Univariate analyses and correlations between chromogranins and clinical data (from all participants or subgroups, as indicated). Bold values correspond to significative p value.

Chromogranins - related parameter	Parameters of interest	Study population involved	Spearman (rho) or Mann Whitney	*p* value
CgA	CST	All participants (n = 100)	0.131	0.193
	CST	Covid+ (n = 73)	-0.183	0.122
	BMI > 30	Covid+ (n = 73)		**<0.05**
	Norepinephrine for MAP over 60 mmHg.	Covid+ (n = 73)		**<0.02**
	Admission albumin concentration	Covid+ (n = 73)	-0.321	**0.0**2
	Lactate	Covid+ ICU+ (n = 49)	0.345	**0.01**
	Creatinine	Covid+ ICU+ (n = 49)	-0.032	0.8
	glycaemia	Covid+ ICU+ (n = 49)	0.284	**<0.05**
	Care-related infections	Covid+ ICU+ (n = 49)	0.347	**<0.02**
	In-hospital mortality	Covid+ (n = 73)		0.682
	In-hospital mortality	Covid+ ICU+ (n = 49)	0.754	0.754
CST	Diabetes	Covid+ (n = 73)		**<0.04**
	In-hospital mortality	Covid+ (n = 73)		**<0.01**
	BMI> 30	Covid+ ICU+ (n = 49)	0.289	**<0.05**
CST/CgA ratio	Gender (ratio male/female)	Covid+ (n = 73)		**<0.02**
	BMI> 30	Covid+ (n = 73)	-0.314	**<0.03**
	Requirement for oxygen to maintain SpO_2_>95%	Covid+ (n = 73)		**<0.05**
	Norepinephrine for MAP over 60 mmHg.	Covid+ ICU+(n = 73)		**<0.04**
	In-hospital mortality	Covid+ ICU- (n = 24)		**<0.01**
	Admission white blood cell count (WBC)	Covid+ ICU- (n = 24)	-0.398	**<0.04**
	Admission lymphocytes (% of WBC)	Covid+ ICU- (n = 24)	0.627	**<0.05**

CgA, chromogranin A.

CST, Catestatin.

BMI, body mass index.

MAP, mean arterial pressure.

To assess any potential prognostic value of both CST and CgA, we plotted data in ROC curves. In the COVID+ patients, with a cut-off value of 2.675 ng/mL, admission plasma CST predicts in-hospital mortality with a sensitivity of 63.2% [38.4-83.7] and a specificity of 75.9% [62.4-86.5] (AUC= 71.25 [58.44; 84.05], *p*<0.01). In the same group, with a cut-off value of 7.97%, the CST/CgA ratio provides an even better prediction of in-hospital mortality with a sensitivity of de 84.2% [60.4-96.6] and a specificity of 57.4% [43.2-70.8] (AUC =69.49 [55.76; 83.23], p = 0.007). In the less severe COVID+ICU- group, with a cut-off value of 1.79 ng/mL, CST predicts in-hospital mortality with a sensitivity of 71.4% [29.0-96.3] and a specificity of 88.2% [63.6-98.5] (AUC = 84.03 [64.61; 100], *p*= 0.002). Finally, in the severest group of COVID+ICU+ patients, with a cut-off value of 12.75 ng/mL, admission CgA predicts the occurrence of care-related infection with a sensitivity of 87.5% [67.6-97.3] and a specificity of 48,0% [27,8-68,7] (AUC=69.42 [54.30; 84.54], *p*=0.015). Admission CgA predicted mortality in neither of the study subgroups.

## Discussion

This exploratory study was designed to determine whether: (i) circulating CgA-derived CST is detectable in COVID patients referred to an emergency department; (ii)) dosages of CST can facilitate understanding unusual inflammatory aspects of the COVID; (iii) CST can of help in treating COVID+ patients requiring oxygen supply.

The aim of the study was to assess CST concentrations in COVID patients as a possible mediator of endogenous inflammation-related pathways triggered by severe infective stress. We programmed dosages to learn whether CST could show the clinical severity of the disease. To enable proper comparison, we defined two groups of controls: healthy ones for with the less severe form of COVID (COVID+ICU-) and COVID-ICU+ patients for the most seriously affected. It emerged that COVID+ patients released approximatively twice as much circulating CST as controls did despite similar circulating amounts of CgA (**Table 1**). Noticeably, COVID+ not admitted to the ICU displayed as much CST as did other patients. Whether this reflects changes in the biological mechanisms of release of granins in this disease is not established; a difference related to the viral load at inclusion was not manifest from our data (**Table 1**). In contrast, the assessment time-window appears important for two reasons. First, on hospital admission CST concentrations had already reached the same value as in ICU+ patients. Second, the accuracy of the CST/CgA ratio in predicting outcome in a mild form of COVID appeared better if the assessment is made early rather than later on ICU arrival.

Previous studies have reported increased CgA concentrations in COVID- ICU+ patients and discussed their origin (27). The absence of acute renal failure in the COVID+ patients in our study suggested the need for additional explanations for increased CgA-release. Hypoxic conditions met in respiratory COVID necessarily augment the release of CST since experimental hypoxia raises that of CgA (30). However, we failed to detect any reliable correlation with either hypoxia-related or even standard blood inflammation-related parameters; moreover, we recorded similar concentrations in non-hypoxemic COVID-ICU+ patients. We therefore hypothesized that CST might be more largely involved in inflammation regulation in the neural regulation of immunity (31).

Humans achieve internal homeostasis during infection by properly balancing pro-inflammatory and anti-inflammatory pathways, including a specific nerve reflex involving the vagus (31). Afferent “sensory” nerve fibres are activated by local cytokines production wherever invasion and injury by infection originate. Signals gathered at the vagal nuclei are transmitted to multiple brain regions for processing. In response, efferent signals arising from the *nucleus ambiguus* and the *dorsal motor nucleus* prevent inflammation from running out of control through acetylcholine (Ach) release at the end of nerves. This rapid-onset connection works in both the *adrenal medulla* and the diffuse neuroendocrine cells within injured tissues. *In vivo*, the vagal reflex is aimed at providing an efficient dampening for any life-threatening pro-inflammatory condition *via* nicotinic receptors (nAChR) (32). The latter are extracellular ligand-gated cation channels activated by the endogenous neurotransmitter Ach or its exogenous analogs (such as nicotine). There is strong scientific evidence that CST acts as a specific, non-competitive, nicotinic cholinergic antagonist of ACh on the first step of the nicotinic cationic signal transduction (33–35). It has been demonstrated that both CST and neuropeptide Y (NPY) have an inhibitory activity on catecholamine secretion induced by nicotine, and that NPY is 10-fold less potent than CST is. CST acts by blocking the access of Na^+^ and Ca^2+^ from the extracellular space to the cytosol, mainly by nicotinic-stimulation, which induces a negative feedback with the blockade of catecholamine secretion. Consequently, CST could affect the vagus efferent signalling by rendering poorly functional the final effector step (36), as suggested recently by the failure of exogenous nicotine to improve outcome in COVID+ patients once admitted to the ICU (37). Furthermore, CST increases the desensitization effect of nicotinic cholinergic agonist-evoked catecholamine release from chromaffin cells (38) which may even re-enforce the impact of the initial afferent pro-inflammatory pathway (34). This amplifies pro-inflammatory consequences by a sustained CST-triggered pro-inflammatory booster on PMNs in combination with its cell-penetrating peptide properties (11). All these data are in line with the negative-outcome prognostic value of circulating calprotectin, which will increase lung injuries (39) in hypoxemic COVID conditions (40, 41). Long-lasting CST-availability contributes to perpetuating an inflammatory vicious circle as long as it hinders neural regulation of inflammation. As shown by its sequences, CST exists in three main genetic variants with loss of potency on the nACh receptor (42). This may explain why populations expressing less potent CST isoforms (43, 44) have a lower death rate from COVID. Finally, *in vivo* deleterious complications may also occur in COVID with other localizations of the nAChRs (45). Typically, during prolonged obstruction or interference with nerve signal propagation from central nervous system stimulation, skeletal muscles will present: (i) fatigue, (ii) disease atrophy, and (iii) sarcopenia (46) persisting until renewal of functionally active nAChRs (45).

Lastly, our data suggest that admission CST could become a biomarker for morbi-mortality. Low CST indicated outcome whether measured alone or when the CST/CgA ratio was taken into account; and prediction was more accurate at emergency department than at ICU admission. However, the reliability of prediction needs validation in multicentre studies, specifically because discrepancies exist on the importance of simultaneous CgA assessment in COVID (18). Our study confirms the reliability of CgA in predicting morbidity issues (ie: care-related infections) but not mortality in line with data in trauma patients (47). This point is of importance because others have reported in COVID a release of Vasostatin-I (the N-terminal domain of CgA) (18), which also impacts inflammation pathways (46).

Our data suggest therefore that when patients’ condition does not deteriorate, and even more when it improves, there is no reason that CTS concentrations increases, since inflammation is meant to decrease over time. As far as ICU admitted patients are concerned, **Figure S1 (Supplementary Data)** shows that CTS concentration first stabilizes and then in a second time tends to increase: these patients are not on recovering as their counterparts in the infectious disease department but will have a more complicated recovery. This indicates that CTS and CgA are released over longer periods of time with possible pharmacological effects *in vivo*. In this setting, they may have new effects *in vivo* hitherto unknown and to be discovered.

In conclusion, human respiratory COVID is associated with significant plasma release of CST, making this molecule largely available for days during this infection. Because of its abilities to both penetrate innate immunity-related cells and to activate the cell-surface nAChR, CST is a mediator shedding new light on immunity in severe human infection. Admission CST and CgA. The original data reported in the present paper are included in the text and the Supplementary Material ; Further inquiries can be directed to the corresponding author assessment could therefore become a biomarker of morbidity-mortality once prospectively validated.

## Data availability statement

The original data reported in the present paper are included in the text and the **Supplementary Material**. Further inquiries can be directed to the corresponding author.

## Ethics statement

The studies involving human participants were reviewed and approved by the Strasbourg University-hospital ethical committee. The patients/participants provided their written informed consent to participate in this study.

## Author contributions

M-HM-B, design of the study and evaluation of Catestatin and Chromogranin A concentrations and revision of the manuscript. FS, design of the clinical study, evaluation and discussion of the results and preparation of the manuscript, PL-B, J-EH, FD, MS, RC-J, VC, recruitment of patients, preparation of plasma and analysis of the clinical parameters. SH and CB, technical assistance for the ELISA assays YG evaluation of the conformity of the ELISA assays. FL, statistical analysis. All authors contributed to the article and approved the submitted version.

## Acknowledgments

The authors thank the University Hospital of Strasbourg and the INSERM as sponsors. FS and M-HM-B thank also Drs Bernard Senger and Naji Kharouf for technical assistance in statistical analyses, and Sylvie L’Hotellier, Michel Masuccio and Céline Picard for data collection, laboratory sampling and study materials provision, and the Association pour la Recherche en Réanimation Médicale, which generously sponsored part of the kits to perform dosages of chromogranin A.

## Conflict of interest

The authors declare that the research was conducted in the absence of any commercial or financial relationships that could be construed as a potential conflict of interest.

## Publisher’s note

All claims expressed in this article are solely those of the authors and do not necessarily represent those of their affiliated organizations, or those of the publisher, the editors and the reviewers. Any product that may be evaluated in this article, or claim that may be made by its manufacturer, is not guaranteed or endorsed by the publisher.
