# Erratum: Assessment of plasma Catestatin in COVID-19 reveals a hitherto unknown inflammatory activity with impact on morbidity-mortality

**DOI:** 10.3389/fimmu.2022.1097810

**Published:** 2022-12-13

**Authors:** Frontiers Production Office

**Affiliations:** Frontiers Media SA, Lausanne, Switzerland

**Keywords:** Innate immunity, COVID, Catestatin, Chromogranin A, hypoxia, critically ill, nosocomial disease

Due to a production error, there was a mistake in **Figure 1** as published. The wrong figure was inserted as **Figure 1**. The corrected **Figure 1** appears below. The publisher apologizes for this mistake.

**Figure 1:**
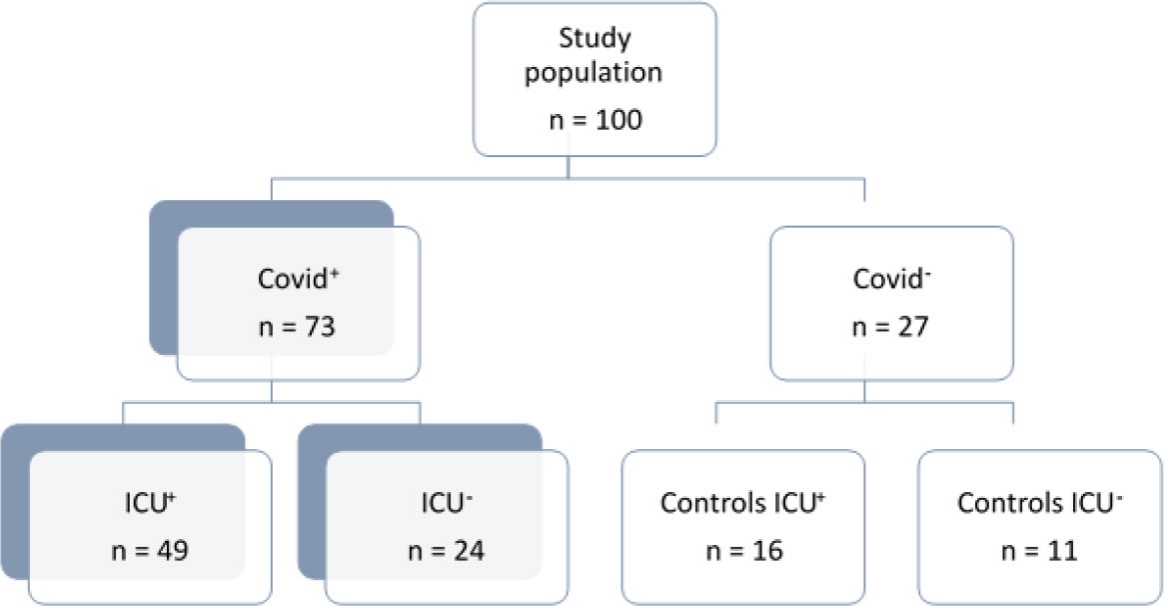
Flow chart of the study. Study participants (n=100) were screened for participation during the first surge of the disease (March to May 2020) among 547 patients admitted for COVID either to the emergency department or to the ICU. Informed consent for participation was obtained in 49 COVID+ ICU+ patients, and in 24 COVID+ICU- patients, which were then admitted to the infectious disease department. In parallel, 11 participants were recruited in our staff (as healthy controls, COVID-ICU-), and so were 16 COVID-ICU+ patients that were admitted for non-COVID multiple organ failure requiring mechanical ventilation support.

