# Supplementary figures and images for: Assessment of plasma Catestatin in COVID-19 reveals a hitherto unknown inflammatory activity with impact on morbidity-mortality
Source: Front Immunol. 2022 Sep 29;13:985472. doi: 10.3389/fimmu.2022.985472 (PMC9559198; doi:10.3389/fimmu.2022.985472)

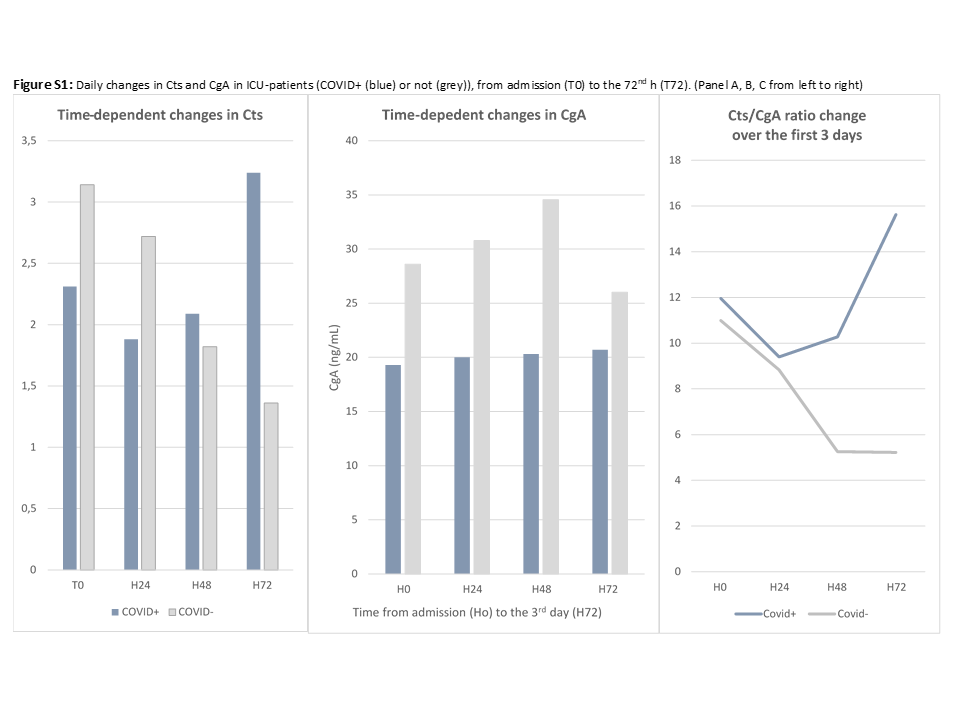

Supplement: Supplementary file 1 [file Image_1.tif]
